# Dr. Baron Ernst von Feuchtersleben on the Principles of Medical Psychology

**Published:** 1848-04-01

**Authors:** 


Art. IV.—*The Principles of Medical Psychology; being the Outlines of a Course of Lectures.* By Baron Ernst Von Feuchtersleben, M.D. Vienna, 1845. Translated from the German by the late H. Evans Lloyd, Esq. Revised and edited by B. G. Babington, M.D., F.R.S. London. Printed for the Sydenham Society, 1847.

There can be no doubt that, generally speaking, very little is known in this country regarding the state of medical psychology in Germany. We therefore gladly hail the appearance of the volume before us, as affording us a fitting occasion for introducing the subject to the notice of our readers.

The present work originally appeared in Vienna in 1845; and we are informed by the English editor, that such was the avidity with which it was sought for, that the publisher found it necessary to recall all those copies which had been distributed to the trade, in order to supply his own customers. We wish that our brethren at home would occasionally exhibit a similar laudable "avidity."

Feuchtersleben is still a comparatively young man. We learn from the preface that he was born in 1809, and took the degree of Doctor of Physic at Vienna, his native town, in 1833. In 1838, he published a work, "On the Dietetics of the Mind," which has now reached its fourth edition; and feeling that the study of psychiatric medicine was much neglected in his own country, he soon afterwards commenced a course of lectures on the subject; and his views were so generally supported, that a new lunatic asylum was built in consequence of his exertions. He is likewise well known as a profound literary character; having published an edition of Hippocrates "On Diet," a continuation of Eble's "History of Medicine," and other works not connected with medicine.

Having thus laid before our readers the claim that our author has upon their attention, we proceed to the consideration of the volume with which the Sydenham Society has favoured us.

In his preface we are told that his primary object has been to write a compendium for a limited class of readers—medical students in a state of transition from theory to practice.

> "He will form a just estimate when he compares the plan with the performance, and bears in mind that what I have written was intended to form the basis of a course of lectures. The work was a mere skeleton, to which symmetry and vitality must be imparted by *viva voce* instruction; indeed the language throughout bears evident traces of the colloquial style, which I have not endeavoured to obliterate, and I therefore beg that the whole may be considered as an outline only of my lectures, which remains to be filled up by future labours."—P. 4.

It is our intention to give an analytical rather than a merely critical review; and, in order that our readers may at a single glance obtain a general idea of the nature of the work, we may observe that (to use the author's words) it separates naturally into three divisions—the first relating to the physiology of the mind; the second, to its pathology; and the third, to the theory of psychological medicine. There is, likewise, an introductory chapter, on the history of medical psychology; and an appendix, on the relation of this subject to regulations for the public health, and to forensic medicine. Before proceeding, however, to the historical department, we must pause for a few minutes over the "preliminary observations." The difficulties of the study of medical psychology are very well described—better, indeed, than we recollect ever to have seen elsewhere; so, also, are the qualifications required in a psychological physician.

The following are those which he regards as imperative:—

"1. To acquire a knowledge of this most delicate and recondite branch of medical science, he must possess, together with that quick apprehension and correct judgment, which are equally indispensable in all the other branches, a refined perspicuity and a philosophically cultivated understanding; for, as we have already intimated, man is not to be understood, even organically, without reference to his higher destination.

"2. The application of this knowledge to the psychological patient requires a very rare and peculiar combination of natural qualities—namely, as much sympathy on the one hand, as firmness capable of assuming the expression of inflexible sternness, on the other. He who is not able to act this twofold part must not be a psychological physician, lest his patients, nay, he himself, should become victims to a mistaken choice of his profession. Besides this, his circumstances must be such as to allow him to devote himself more or less exclusively to this branch of medicine, that is, to give it the greater portion of his time, which is more necessary in this than in other branches, because the treatment in most instances demands a second education. He must be able by his personal demeanour to obtain influence over the minds of other men, which, though in fact an essential part of the psychical mode of cure, is a gift that nature often refuses to the most distinguished men, and yet without which mental diseases, however thoroughly understood, cannot be successfully treated.

"Lastly. He ought to possess a high moral character, which, indeed, no person should be without in any branch of science, but which is here especially needed. . . . Let every one, therefore, sincerely and carefully examine his conscience, his powers, and his wishes, before he devotes himself to this most difficult of all the departments of medicine; for more confusion has been produced in the world by careless and incomplete efforts than by total inertness."—P. 22.

The historical chapter, to which we have already referred, occupies upwards of fifty pages. As a plea for the extent of this portion of the work, our author very correctly urges that that part of medical psychology "which is philosophical, contains an abstract of the state of philosophy in every age, while that which is empirical has by no means attained such precision and clearness as to render a knowledge of previous opinions superfluous; on the contrary, even at this moment, the opinions of some are diametrically opposed to those of others."—Pp. 23-4.

The whole history is divided into four heads:—

I. The primæval and pre-historical epoch to the development of the sciences in Greece.

II. The ancient epoch—the time of the Greeks and Romans.

III. The medieval epoch, divisible into (*a*) the scholastic, which accompanies the decline of ancient art and science, and (*b*) the revival; and,

IV. The new epoch, comprehending the present state of the sciences as a whole.

In each of these epochs the author considers—

1. The general character of the age, as far as it bears on the subject.
2. The history of the health and disease of the age, sketched with a psychical view.
3. The history of the science—*i. e.* of the theory.

4. That of the art—*i. e.* of practice; and,

5. A critical recapitulation with respect to each epoch.

Our information regarding the first epoch is, as might be expected, scanty in the extreme. Psychical sufferings, says our author, must have been as rare in that infant state of society, as they are in the infancy of every individual man. Reference is made to the few cases of which any record has been handed down to us. Saul's disorder is regarded as a combination of melancholy with rage; while Nebuchadnezzar's bore a resemblance to lycanthropy—nay, even to pellagra. "Many of the narrations recorded in the Old Testament, and some of the δαιμονίζομενοι in the New, are ascribed to madness. Greek mythology, in the stories of Hercules, Ajax, Orestes, Athamas, and Alcmæon, touches on these phenomena, and on lycanthropy; and the madness of the daughters of Prœtus, and the uterine disease of the Scythians, are even quoted as examples of epidemical psychopathies." It is no less singular than interesting to find that some of the first cases on record were psychical; as, for instance, the cure of Saul by music; and that of the Prœtides, which was effected by Melampus, partly by psychical, and partly by ordinary means.

We pass onwards to the second epoch. The general character of the age is exquisitely depicted. There is a force and elegance in the description that almost tempts us to extract it, as affording a model for all future translators. But we must refrain; and shall, in its place, give the following sketch of the general psychical conditions of that period.

"It is susceptible of proof, that with the increase of refinement the occurrence of nervous and mental disorders increased in a proportion which has been maintained to the present day. So long as Greek heroism continued to echo the natural simplicity of the Homeric age, so long as the unsophisticated manners of the old Romans subsisted, there was no occasion to notice the occurrence of such diseases. With the advance of civilization in Hellas they appeared now and then, though in truth but rarely. But so soon as civilization degenerated into voluptuousness, they increased in number and intensity; and when at Rome, unbridled debauchery and insane luxury surpassed even the pomp of Athens, from which the Graces had not wholly departed, then did those psychical anomalies increase, and such in particular as are frequently mentioned in Galen's work on Diseases of the Mind. The monomania for suicide of the Milesian maidens, and the feverish psychical excitement of the inhabitants of Abdera after witnessing the performance of the Andromache of Euripides, are adduced as being in some degree instances of an epidemic psychopathy."—P. 27.

Let us now examine the "practical psychiatrics" of that period. Of the Greek philosophers, Pythagoras was the first who attempted the separation of medicine from priestcraft; "who led it forth from the temples into active life, applied it to political economy and legislation, directed his especial attention to dietetics, and who, lastly, must be considered and honoured as the true founder of a system of psychical dietetics." According to Seneca, he employed music in the cure of those chronic diseases which originated in mental excitement. In the writings of Hippocrates we find more references to the psychical symptoms occurring in various diseases than to what we specifically term mental diseases. He notices the physical insensibility of the insane; the appearance of mental diseases in spring; the occurrence of disorder of the intellect after prolonged fear and grief; the union of melancholy and epilepsy; the critical importance of hæmorrhoidal discharges in mania; and the difficulty of curing madness which commences after the age of forty. His treatment consisted for the most part in evacuation; and the drug on which he principally relied was hellebore.

The real founder of a psychical mode of cure seems, however, to have been Asclepiades, (born about 90 B.C.) It is reported (for none of his writings are extant) that his principal remedies in the treatment of the insane were music, love, wine, employment, exercising the memory, and fixing the attention. He was strongly opposed to bodily restraint, except in the most dangerous cases.

"He was peculiar," says our author, "in advising that the lunatic patient should be engaged in the self-regulation of the mental powers, for which purpose he recommended that books should be read to him in an inaccurate manner, that he might be induced to correct the mistakes. The more exact diagnosis of improved science discovers cases in which a similar mode of proceeding is often useful; and it is usual to modify it by placing before the patient, without apparent design, letters, essays, notes, &c., composed by others, for him to correct, whereby his own energy is increased and confirmed."—Pp. 36-7.

Celsus, a sort of Roman Copland, presents us with the first treatise on mental diseases, in his chapter, *De Tribus Insaniæ Generibus*, which probably makes up in a great degree for the loss of most of the earlier writings. Feuchtersleben notices the labours of Celsus in the following terms:—

"With judicious criticism, scrupulous completeness, and incomparable arrangement, Celsus gives a compressed recapitulation of all which up to that period had proved to be best and most correct; and where no precise result had been obtained, he states, pointedly and faithfully, such problematical views as were entertained. He distinguishes three kinds of insania: phrenitis, which is a continued delirium; melancholy, which originates in an atrabilarian condition; and a third kind, which lasts longer than the other two, even sometimes through the whole course of life, and which may be divided into species, according as the patient is deluded by merely false images, or by erroneous perception. The first and the last of these kinds of insanity are sometimes of a melancholy cast, sometimes cheerful, by which the psychical treatment is modified; the second is always melancholy. Phrenitis is treated according to individual indications; melancholy requires the evacuant and sedative method. In the third kind, when it approaches more to melancholy, it requires purgatives, and when to phrenitis, emetics. Exercise, friction, and an unirritating diet must be resorted to, and psychical influence must never be forgotten. The further details of this treatise are highly worthy of attention."—Pp. 37-8.

Thessalus of Tralles, although "a man whom history represents as an ignorant, boastful, low-minded pretender, a charlatan in the worst sense of the word," requires a brief, passing notice, as being the inventor of metasyncrisis (recorporatio); a method which sets forth a most important corporeal means in the treatment of insanity, and to which we shall return in a future part of this article.

Aretæus, in the fifth and sixth chapters of his book on the "Causes and Symptoms of Chronic Diseases," gives a correct and lucid description of melancholy and mania. Amongst other varieties of mania, we have a distinct sketch of religious monomania.

"His therapeutics," says our author, "were those that had been handed down to him from former times. Portal attaches particular value to one observation which Aretæus makes on this occasion, respecting the pernicious influence of coloured walls in the chambers of the insane, and confirms it by his own experience. In fact, every one who has observed the effects of colour on the mind, and is acquainted with the doctrine of hallucination, may be easily convinced of the truth of that observation."—P. 39.

Cælius Aurelianus is dismissed in four lines; and of Galenus (with whom the ancient epoch concludes) it is observed that, "we find in his numerous works fewer psychiatric precepts than we should have expected, and they confirm in essential particulars the doctrines already handed down."

We enter a new epoch—the medieval period; an age in which all nations were in commotion, and when "the most diverse religions, modes of life, views, and traditions, clashed and blended together." The psychical momentum influencing the dancing mania, is alluded to; but our author does not go so far as Wawruch, "who treats even the crusades as an epidemic mental disease." Webster describes an epidemic madness which prevailed in England in 1354, attacked the lower orders, and subsequently spread through France and Italy.

Little was achieved for psychological medicine during this period, even during the latter portion of it, from Paracelsus downwards:—

"The practice of medical psychology," says Feuchtersleben, "was upon a par with its theory; that is to say, it did not exist at all during the greater part of this epoch. The Arabs repeated and followed, with less and less happy additions, and with more or less genius, what had been taught by the Greeks, especially the later writers..... Ibn Sina, the Persian, as the best of these writers, deserves, however, distinct mention. He paints to the life the nymphomania arising from unhappy, unrequited love, adding some original reflections of his own; and he gives a contrivance for the cure of melancholy, which has much resemblance to our swing."—P. 45.

The modern epoch now claims our attention. It is, however, so extensive and important, that our author subdivides it, detaching from it the present century. In his remarks on the history of health and disease during that period, he notices a subject that has never yet, so far as we are aware, met with the consideration it deserves. It is the difference of type that diseases present at different periods. During the first half of the period now under consideration, a more than usually vigorous state of health predominated, usually demanding antiphlogistic measures:—

"With the commencement of those storms in social life, by which many a victim was immolated, many a peaceful existence destroyed, an asthenic state of the general health visibly set in, and manifested itself at first chiefly in the vascular system . . . but it gradually affected the roots of life more deeply, and fixed itself in the nervous system. This nervous character is that of the present day, for neuroses of every form have become more and more developed, especially through the middle of the eighteenth century. At the beginning of the eighth decennium, raphania, which often commenced with mania and terminated in imbecility, became particularly prevalent. In the closest connexion with these neuroses, are mental diseases, which, in a psychical point of view, are fostered by an education calculated more for the world than for the formation of character, and in a somatic point of view, by that condition, of such frequent occurrence, known by the name of abdominal plethora. In fact, the nearer we approach to the present time, the more manifestly mental disorders increase."—P. 51.

Amongst the names to which, during this epoch, psychology as a science is the most indebted, we may mention those of Locke, Condillac, Bonnet, Leibnitz, Wolf, and Kant: in the practical department, we must especially insist on the claims of G. E. Stahl, who may be said to have been the first who united psychology with practical medicine, and of the fellow-labourers, Reil and Hoffbauer, and of Pinel, in France, and Chiarugi in Italy.

The following sketch of the principal asylums of Europe will be read with interest:—

"Paris was distinguished by the noble institutions of the Bicêtre, for 800 male lunatics, and the Salpêtrière for as many females, which, with their dependencies, present the appearance of a little town. Besides these, the institution at Charenton, near Paris, and the admirable one at Rouen, are well known. In England, the fine asylum at Hanwell, for pauper lunatics of the county of Middlesex, which accommodates 1000 patients, the splendid New Bethlehem, and St. Luke's hospitals are the most distinguished. In Italy, there are noble establishments—namely, in Genoa, Ancona, Aversa, and at Palermo, under the direction of the philanthropic Baron Pisani. The establishment at St. Petersburgh has become eminently useful. In Switzerland, great service has been rendered by Dr. Tribolet, near Berne. Spain, torn by intestine troubles, is unfortunately by far the most backward nation in this respect. In Germany, the opening of the Sonnenstein, near Pirna, in Saxony, was the dawn of a day which was thenceforth to cheer the most unfortunate of the human race. The able and energetic Langermann came from Baireuth to Berlin, to regulate this institution, and to superintend the organization of other lunatic asylums. The establishment connected with the Hôpital de la Charité, in that city, now directed by Dr. Ideler, and the private institution of Horn, soon acquired deserved reputation. The other celebrated institutions in Germany are—in Halle, (now under Damerow;) in Marsberg, for Westphalia, (Ruer;) in Siegburg, for Rhenish Prussia, (Jacobi;) in Würzburgh, united with the Julius hospital, (Narr;) in Münich, (Christmüller;) in Leipzig, where Heinroth practised, near Achern, (Roller;) in Merxhausen, in Hesse, (Gross;) in Hofhein, likewise in Hesse, (Amelung;) in Winnenthal, (Zeller.) A splendid new building, which is not yet inhabited, has been erected at Erlangen; and Saxenberg, near Schwerin, offers the first model of a great establishment in which the treatment of the curable and the care of the incurable are combined. The principal institutions in the Austrian empire are—in Vienna, (Dr. Viszänik;) in Prague, (Dr. Riedel;) in Gratz, (Dr. Schwbert;) in Brünn, (Dr. Kroczak;) in Laibach, (Dr. Zhuber;) in Clagenfurt, (Dr. Jansekowich;) in Hall, (Dr. Tschallener.) In conclusion, we have still to notice the remarkable colony for the insane at Gheel, near Antwerp, where between 400 and 500 lunatics are distributed for their cure among the 6000 inhabitants of the place; and the establishment of the philanthropic Dr. Guggenbühl, on the Abendberg, in Switzerland, for the cure of cretinism. Even in Egypt, Mehemet Ali has, under the direction of a European physician, appropriated the civil hospital, Esbekieh, to the reception of the insane, who had hitherto languished in that country in a state of helpless destitution."—Pp. 66-7.

The chapter concludes with a preliminary idea of the present doctrine of the science. Our author notices the three views of the relations of intellectual to corporeal life maintained by the writers of the present day, namely:—1. The *somatic*, which assumes the operations of the mind to be an emanation from those of the body, and considers mental disorders to be merely bodily ailments; 2. The *psychical*, which assumes an independent operation of the mind, and considers its disorders as purely psychical derangements; and, 3. The *mixed*, which assumes an independent operation (life) of the mind, and sees in its derangements a half-psychical, half-corporeal disease.

His own opinions seem to lean towards the last view; but, as he very sensibly remarks, the importance of these theories is by no means so great in its application as might at first sight appear.

"As in the healing art in general, the most experienced and best informed physicians pursue nearly the same treatment at the bedside, and only explain in a different way the effect of the same remedies, according to the difference of the schools to which they belong, so those who practise this branch of medicine in particular agree on the whole in the choice of remedies; the psychical employ likewise somatic; the somatic employ likewise psychical remedies; only, as we have seen above, the one party explain the effect of the psychical remedies somatically, and the other the effect of the somatic remedies psychically. . . . . All practical men in this branch are, however, especially unanimous in acknowledging the importance of public institutions for the cure of lunatic patients; all eyes are directed to them, and plans for their amusement and improvement are now the primary object of psychiatric science; and though the sanguine admirers of the psychical mode of cure, for instance, by theatrical representations, &c., undoubtedly go somewhat too far, it is nevertheless generally perceived that these institutions and their proper organization offer the only practicable mode of successfully treating mental disorders."—P. 74.

We proceed to the consideration of the physiology of psychiatrics: but we shall not follow our author so closely through this portion of his work as we have previously done, or as we shall do in the succeeding parts. It belongs more to metaphysics than to medicine, and does not admit of satisfactory condensation.

The effects of the passions are forcibly described:—

"Who," says our author, "does not know the constant excitement, fluctuating between pleasure and pain, in which love keeps the body and mind? A continual state of abstraction, now and then interrupted by deep sighs, and a change in the temper and habits, betray its commencement; change of colour, of expression, of the pulse, denote its object. The pale countenance, the languid eye, the low pulse, want of sleep, declining bodily health, its loss or its hopelessness; the flushed cheek, the brilliant expression, the accelerated pulse and breath, increased vital turgor, plainly indicate its happiness and bliss. That all these phenomena are much more evident in the female sex than in the male, arises physically from their more delicate conformation, and psychically from the higher value which they attach to love, as the proper business of their lives."—P. 141.

Again, in regard to anger, after quoting Seneca's well-known description of its effects, he observes that:—

"The clonic spasm of the muscles, which manifests itself in tremulous motions, indicates the struggle in the conflicting excitement; this excitement urges the circulation to the utmost vehemence; the respiration keeps equal pace with it, so that in the most violent cases, bursting of the heart, and even pneumothorax take place. It acts, through the vegetative nervous system, upon the secretions, the saliva, the milk, and the bile, which often become actually poisoned. Tourtüal saw a child die, as if struck with lightning, after taking the milk of its enraged nurse. Whether hydrophobia can be induced by the bite of an enraged man is still undecided; but that bilious diseases may be brought on by passion, is well known. On the other hand, more rarely indeed, the vital energy, excited by anger, overcomes obstinate organic obstructions, contractions, paralysis, &c. These facts have given rise to the notion of assigning to each passion its particular relation to certain organs of the body—love to the heart, anger to the liver, &c.; relations which, as we have seen, certainly exist, but which find a more general explanation in the mutual dependence of the organisation, and in the constitution of each individual."—P. 142.

The functions of the nerves, brain, and ganglionic system, the differences between men—as dependent on race, temperament, occupation, education, &c.—physiognomy, cranioscopy, sleep, dreaming, intoxication, and vertigo, are amongst the subjects treated of under the head of *Physiology*.

We proceed to the consideration of *the morbific influence of the body on the mind, and of the mind upon the body*.

In attempting to show *how* the blood affects the action of the nerves, and thus acquires psychical importance, he notices the effects of transfusion in cases of debility or anæmia; and of vascular repletion, increased plasticity, and predominant arterial action, in heightening, to a greater or less degree, the psychical affections and reactions; and the contrary effect of an empty or relaxed condition of the vessels, in depressing the nervous power, and producing the same sinking tone of mind as is experienced after venesection and considerable loss of blood.

"The same result (he adds) is produced by diminished plasticity and preponderant venous action. It is certain that a corrupt state of the blood, whether it arise from a sporadic, miasmatic, contagious, or any other cause, has the effect of depressing the spirits. The spleen of the English is partly attributed to an endemic influence of this kind, which corrupts the blood through the atmosphere. Amongst the dyscrasies, the gout especially acts on the mind through the brain. Among the cachexies it is the scurvy. This latter and melancholy reciprocally promote each other.

"Organic lesions of the heart and great vessels, whether originating in hypertrophy, torpidity, ossification, enlargement, or any other cause, produce a feeling of discomfort, anxiety, and lowness of spirits. Ossification of the internal coat of the arteries, and especially of the heart, are [is] said to be accompanied by a certain phlegmatic tone of mind; and Klencke connects the endemic phlegma in England with the endemic ossifications of these membranes which occur in that country."—P. 176.

Alas! for poor England, with her endemic corrupting influences, and her endemic ossifications! We know nothing of Herr Klencke, further than that he is the author of a *system of organic psychology*, published some six years ago, which is characterized by Feuchtersleben as one "of the most reputed hypothetical and symbolical essays of the kind."

The effect of certain medicines in modifying the blood, and thus influencing the mind, are, we think, undoubted. A dose of nitre or digitalis can convert cheerfulness into low spirits, while tincture of cinnamon and other medicines exert an opposite effect.

The functions of the lungs, skin, and stomach, each and all, exert a similar influence on the mind. We might quote almost innumerable examples of the action of the respiratory on the psychical functions, both for good and for evil. Who has not experienced the cheering and vivifying influences of the mountain breeze, or witnessed the yet more striking effect of certain gases, inducing "a sort of ethereal intoxication?" Look at the care-worn bookworm. The close air of his badly-ventilated study, and the impeded respiration dependent on the absence of exercise, too often render him a gloomy hypochondriac, a misery to himself, a burden to all around him.

In the remarks on the influence exerted by the function of digestion on the mind, we meet with the following very singular observation:—

"The changes are equally well known which the temper suffers through the regularity or obstruction of the normal excretions. Could we penetrate into the secret foundations of human events, we should frequently find the misfortunes of one man caused by the intestines of another, whom the former endeavoured to inspire with sympathy in his fate, at a moment when the frame of mind of the latter was affected by impeded secretion. An hour later, and his fortune would have been made."—P. 179.

Which, we presume, means in plain English, *Don't ask a favour of your friend when he looks bilious.*

The effect of ileotyphus fever on the brain is noticed by our author under this head.

The influence of the sexual function on mental life next claims our attention; and we rejoice to find that our author maintains much the same view with ourselves as regards the assumed evil consequences of chastity:—

"The act of coition itself has a decidedly psychical effect. If exercised with moderation, at full maturity, and at the right moment, it leaves (notwithstanding the '*omne*

*animal post coitum triste,'*) a pleasurable feeling; nay, it invigorates the powers of thought, as is shown by the example of the ingenious voluptuary Casanova, who at such moments solved the most difficult mathematical problems. If not gratified when urgent desire exists, it may indeed occasion psychical uneasiness, and especially distract the attention; but as the corporeal ill consequences of abstinence have always been estimated much too highly, so also a cultivated understanding and a vigorous will, will not have, psychically, much to suffer from them. If inordinately indulged, it leaves, through the exhaustion of the nervous power, a sensation of mental depression, and if too often repeated, total debility of every mental power."—P. 181.

The various influences of menstruation, pregnancy, lactation, and the climacteric change, the influence exerted by the abnormal proportion of individual structures, by the weather, diet, and various cosmical agents, are all duly noticed, as also are the influences of feeling in its various manifestations, and of the continued excitement of the organ of the will (the motor nerves.) But we must proceed to a new department—the semeiotics of our subject. And here we meet with some very important practical matter, which we will endeavour to lay in a somewhat condensed form before our readers.

From intense anxiety and grief, we may be led to expect organic affections of the heart and larger vessels; while fretfulness, dejection, and discontent, point to a deranged state of the digestive organs. General want of memory, or rather, difficulty of remembering, is often a symptom of hyperæmia of the brain, and therefore a premonitory symptom of apoplexy. If it occur in the course of acute diseases, it is a symptom of sopor or of delirium, and precedes cerebral inflammation and softening, and the eruption of violent exanthemata; while in chronic cases it indicates congestion and oppression of the brain.

Anamnesia, or loss of memory, if it occur in acute disorders, generally betokens a fatal termination, if not an instantaneous crisis; while in chronic diseases it usually indicates incurability, or when it occurs suddenly, in epileptic and hysterical patients, the immediate approach of a paroxysm.

Moroseness is a symptom of determination of blood to the brain. Dreaming has, according to our author, very frequently a semeiotic indication. We doubt, however, whether all the opinions given in the following quotation, can be regarded as fully established:—

"Dreaming, as the precursor and accompaniment of diseases, deserves continued investigation, not because it is to be considered as a spiritual divination, but because, as the unconscious language of the cœnæsthesis and of the *sensorium commune*, it often very clearly shows to those who can comprehend its meaning, the state of the patient, though he himself is not aware of this; and the interpretation of dreams deserves the attention and study of the physician, if not of any one else . . . Albers sets forth the following signs as the most approved:—

"Lively dreams are in general a sign of the excitement of nervous action.

"Soft dreams are a sign of slight irritation of the brain; often in nervous fevers, announcing the approach of a favourable crisis.

"Frightful dreams are a sign of determination of blood to the head.

"Dreams about fire are, in women, signs of an impending hæmorrhage.

"Dreams about blood and red objects are signs of inflammatory conditions.

"Dreams about rain and water are often signs of diseased mucous membranes and dropsy.

"Dreams of distorted forms are frequently a sign of abdominal obstructions and disorders of the liver.

"Dreams in which the patient sees any part of the body especially suffering, indicate disease of that part.

"Dreams about death often precede apoplexy, which is connected with determination of blood to the head.

"The nightmare, (*incubus, ephialtes,*) with great sensitiveness, is a sign of determination of blood to the chest.

"We may add that dreams of dogs, after the bite of a mad dog, often precede the appearance of hydrophobia, but may be only the consequences of excited imagination."—Pp. 197-8.

We proceed, without further remark or extract, to the second great division of our subject, its pathology.

The first subject treated of under the pathological division is sleep-walking, or idio-somnambulism, the phenomena of which are very lucidly described. Our author regards the disposition to somnambulise as depending "somatically, on a delicate nervous system, and, psychically, on a predominance of the direction of fancy over that of intelligence." It is often hereditary; belongs more to the female than to the male sex, to youth than to mature age, and is especially manifested at the age of puberty.

The most frequent somatic causes are excessive fatigue, immoderate sexual intercourse, intemperance, and various diseased conditions of the system, such as menstrual anomalies, gastric or abdominal irritation, hysteria, and possibly structural disease of the brain; while on the psychical side may be mentioned, "acute mental sufferings, profound grief, excessive tension of the intellectual powers, passion—all which, with the above-mentioned psychical disposition, may be promoted by a too effeminate education." Although sleep-walking is often transitory, and yields readily to treatment, cases which defy every remedy sometimes fall under the notice of the physician, and they should be regarded with suspicion:—

"In bad cases," says our author, "incurable mental diseases have frequently been observed as maladies resulting from it; and thus experience indicates this state as a pathological transition to the psychopathies. . . . Frightful dreams generally trouble the short sleep of the lunatic."—P. 205.

Passing over the subject, of delirium in its various forms, hyperæsthesia, cœnæsthesia, pseudæsthesia, satyriasis, nymphomania, and hypochondriasis, we must pause on arriving at hysteria. Feuchtersleben is a bold man, and does not fear to speak his thoughts plainly. Is there a single practitioner in this country who in his heart does not fully concur in the following observations regarding the exciting causes of this disease? Yet how often do we fail in our duty in impressing these views on the heads of families and on society at large!

"Those," says our author, "who have seriously contemplated the female education of our times, (undoubtedly the *partie honteuse* of the moderns,) will find it, in this etiological respect, much more influential than that of the other sex. It combines every thing that can heighten sensibility, weaken spontaneity, give a preponderance to the sexual sphere, and sanction the feelings and impulses that relate to it. This is not the place to discuss these positions, which are well worthy of examination. To those who are acquainted with the state of society, they are self-evident. On the somatic side, disordered states of the sexual system are here particularly to be kept in view, such as result from irregular menstruation, impotent husbands, and frequent child bed confinements. The excessive use of coffee and tea undoubtedly deserves also especial mention."—Pp. 228-9.

In a brief sketch which our author gives of the principal outlines of the general pathology of the psychopathies we find some interesting remarks on their epidemic occurrence. Our classical reader will recollect the description given by Herodotus of such an epidemic among the Argive women, which began with the daughters of Prœtus. They ran into the woods and murdered their own children, but were cured by Melampus, with *veratrum album.* The Milesian maidens, as Plutarch tells us, took to hanging themselves, and the monomania "was cured psychically by a law, that the bodies of those who had thus destroyed themselves should be exposed naked." The madness of the Abderites described by Lucian, which was consequent on the representation of Andromache, and was possibly not altogether unlike the Jenny Lindism of the past summer, probably belonged to the same category. The Scythians often laboured under the fixed delusion that they were women. Among the Greeks lycanthropy sometimes appeared as an endemic.

"In Arcadia, a country abounding in forests, morasses, and pasture land, the fixed delusion of being a wolf frequently arose among the shepherds, and was accompanied by conduct suitable to the character of that animal. A similar disorder occurs amongst the aborigines of Brazil. After the Indian has wandered about for a time, pale, silent, reserved, with a confused, fixed stare, he suddenly breaks loose in the evening, after sunset, runs raving through the village, howls, turns up the graves, and rushes into the woods. The attack terminates in exhaustion, or passes into fever."

The dancing mania in the middle ages, the epidemic madness which prevailed in this country in 1354, and various *extases religiosæ,* afford other and better authenticated illustrations of epidemic insanity.

Our information on the *geography of psychopathies* is very vague and uncertain, for reasons which will be sufficiently obvious to most of our readers. On the subject our author remarks that :—

"Except, perhaps, Turkey and Egypt, insanity is more rare in other quarters of the globe than in Europe; but, according to Brigham, it is more frequent in civilized America than in Europe; among savages it is almost as rare as among children. In Russia it is very frequent, particularly in the form of mania; whereas the Finnlanders are more subject to idiocy. In France, it increased very much after the Revolution, but since 1830 it has again decreased. In Great Britain, the country where originality has been carried even to eccentricity, the number of lunatics in 1826 amounted to 8000; in France, to 3000; in Holland, to a proportionably greater number; in Prussia, the proportion is given as 1·666; in Norway, as 1·551. Italy gives a particularly favourable proportion. A difference is likewise to be observed in the occurrence of several forms. In the south, mania is most common; in the north, melancholy; in the valleys, idiocy, &c.; in England, fixed delusion predominates; in France, fatuity; in the East, idiocy; Germany presents a more happy medium."—P. 254.

Happy Germans, quietly and philosophically rejoicing in a medium of mania, melancholy, idiocy, fixed delusion, and fatuity!

The critical processes under which psychoses frequently disappear, are arranged by our author under the two following heads:—

1. The return of suppressed excretions and secretions; and,

2. The return of previously existing morbid deposits or affections, as of psora, herpes, discharging ulcers, hemicrania, &c.

In relation to the excretions, he regards the skin and the bowels as the organs most commonly giving relief; the kidneys more rarely, and the

Schneiderian membrane and the salivary glands, very seldom. When the venous circulation is impeded, an eruption of furunculi may cause the psychopathy to disappear.

Considerable space is devoted to the necroscopic appearances presented by the insane; but we regret to say that our author, like almost all preceding writers, arrives at no very definite conclusion. He truly observes—and it is a point that some of our best writers have overlooked—that "the individual organic metamorphoses are not the disease itself." He points out the special necessity in this case of distinguishing between the *cum*, the *post*, and the *propter*; "for necroscopy in diseases of personality is only so far instructive, as it points out to us in these conditions everything that co-operates and is operated on organically." Our investigation should not merely extend to the brain but to all the organic structures, and should include their chemical composition.

The dimensions of the head are generally diminished in idiocy, but enlarged in the other forms of the psychoses. The cranium is generally very thick. The clinoid processes are often elongated and sharp, and elevations frequently occur on the base of the skull. According to Kasloff, the foramen lacerum posterius is always contracted, whence he concludes that the impeded return of venous blood is the cause of insanity. The pia mater and arachnoid often present a thickened appearance, and are studded with small, white, soft masses of exudation. Fluid is often effused between the meninges, particularly in patients who have been raving mad. Hyperæmia of the cortical layer of the brain, especially in furious patients, is very constant, and our author seems to incline to the well-known opinion of Ronberg, that "on the psychoses, the substance of the brain is much more rarely altered than the vascular system."

Chemistry has as yet thrown little light upon the alterations induced by the various forms of insanity on the composition of the brain. The following are the changes observed in other parts of the system:—

"Adhesion of the pericardium to the heart and to the pleura, water in the pericardium, and ossification of that membrane, are not uncommon appearances in the bodies of insane persons. The size of the heart, especially in furious maniacs, has been more frequently found to be diminished than enlarged; the heart itself, particularly in melancholy patients, is often flabby and soft. Thickening and ossification of the valves are of frequent occurrence.

"The blood appears for the most part thicker than in healthy persons, black, and disposed to polypous coagulation, but also very frequently thin and watery.

"Tubercles in the lungs are well known to be very common in the bodies of insane persons.

"Things of various kinds which have been improperly swallowed are found in the stomach. In some cases mentioned by Greding, the position of the stomach was abnormal, as well as that of the intestines. Wichmann declares contraction of the large intestines to be a diagnostic sign of insanity.

"Affections of the liver are so common in the insane, that Cheyne assures us Lood found the liver diseased in 400 bodies.

"The spleen is likewise generally diseased, often enlarged to the weight of several pounds, (in one instance, thirty pounds.)

"The gall-bladder has been often found adhering to the duodenum and the eleum.

"These are the more general, more important, and more constant results of psychiatric necroscopy."—P. 259.

These investigations are much too isolated; and the observers have, unfortunately, too often neglected to indicate the form of insanity, or the stage at which death ensued, to enable us to draw any certain conclusions. Moreover, it must be regarded as an established fact, that the intensity of the disturbance of the functions does not necessarily stand in any fixed relation to the change of structure of the organs.

We regret that our allotted limits preclude us from following our author at any length through the remainder of this chapter. We cannot, however, refrain from briefly noticing the "causes of insanity," and "the natural description of the forms of mental disorder as they really appear."

In reference to the general pathogeny of the psychoses, we must discriminate between the predisposing, occasional, and proximate causes.

### PREDISPOSING CAUSES.

1. *Hereditary descent* is unquestionably the most frequent predisposing cause—more than half of all the cases that occur being occasioned or favoured by it. The danger is less when the procreator does not become insane till after the procreation.

2. *Psychical and physical temperament.* The tendency is exhibited (*a.*) psychically, by passiveness in thinking, in feeling, and in willing; (*b.*) physically, by predominant erethistic vital debility, the fundamental character of the present generation. We must also take into account certain constitutional diatheses on the corporeal side—namely,

(*α.*) The scrofulous and rachitic *habitus,* in which the above-mentioned state occurs.

(*β.*) The apoplectic, which in consequence of the hyperæmia of the brain predisposes to mania ; and—

(*γ.*) The venous or atrabiliary, which predisposes to melancholy, in consequence of the impeded ganglionic conduction.

3. *Sex.*—On this point no general law can be laid down. The intellectual and physical national character, the position of the female sex, and local circumstances, determining the result.

4. *Age.*—The cycle from sexual evolution to sexual involution is that in which mental disease most commonly shows itself. Cases occurring before the years of puberty are very rare. Our author observed a case of moria in a girl six years old. Ideler, in noticing cases of rare psychoses occurring after the epoch of involution, mentions having had to treat women seventy years old for erotic madness. Esquirol cured two women, each eighty years old.

5. *Education* must undoubtedly be placed high among the predisposing causes. Education, says Feuchtersleben,—

> "When judiciously and energetically directed, can raise a barrier against the strongest, even the hereditary tendency. Thus by unbridling or judiciously restraining the fancy, it may either encourage or check this unhappy predisposition. We see, therefore, from this, also, how far we have to carry back our recollection of prominent circumstances in the investigation of psychopathic diseases. But if our over-refined education is to be considered as promoting this disposition, all seems to depend on the notion which is attached to this assertion. Education can never be carried too far, so long as it is consistent, and the higher it is carried in this sense, the more protection will it afford on the psychical side against the invasion of insanity."

6. *Employments* are noticed among the predisposing causes, especially those of the weaver, shoemaker, and worker in metals. Here, however, we have a commingling of occasional and predisposing causes.

OCCASIONAL CAUSES.

These are divisible into *psychical* and *physical*.

The principal psychical causes are—

1. Neglected cultivation of the mind, and idleness.

2. Partial cultivation of the mind in one direction, especially in that of the fancy.

3. Emotions and passions; especially ambition in men, and love in women.

The consideration of these three causes furnishes us with a clue to the elucidation of the question why the number of mental diseases has increased with civilization.

"It is not civilization," says our author, "but the increasing work which it brings in its train; partial education, passions, emotions, &c., all which set the mind in passive motion; the forced culture to which they lead; the over-indulgence; these contain the reasons of this fact. Civilization, as external education, is but a transition to culture as internal education; and in this first stage it produces evils for which it furnishes the remedy in the higher stages. It carries the poison and the antidote in the same hand. The industrial impulse of the present time, for instance, by the hazards to which it exposes the opulent classes, is one of the occasioning momenta, while by the activity which it excites, and by doing away with isolation, it is one of those which is counteracting and salutary. If savages show such a happy exemption from insanity, they are indebted for it, not merely to the want of civilization, but probably also to the indomitable energy of their corporeal vitality."

The principal physical causes are—

1. The extremes of cold and heat—the former of which may cause obtuseness of intellect, the latter furious mania.

2. Different states of the atmosphere, which in towns seem favourable to melancholy; on the mountains, to excitement; and in the valleys, to idiocy.

3. Traumatic influences, not only immediately on the head and brain, but in any region of the nervous system.

4. Animal and narcotic poisons; as, for instance, *virus hydrophobicum*, *serpentum*, &c., *cicuta*, *stramonium*, *agaricus muscarius*, &c. Under this head we must also place spirituous liquors.

5. Purely somatic morbid processes, which, by causing abnormal irritation of the nerves, may occasionally give rise to psychical processes; amongst these we may especially mention itch, biliary and urinary calculi, heart and liver disease, and gout.

6. Many observers, says our author, maintain, while others deny, that the moon and its phases have an exciting influence on the exacerbations and fits in psychical patients.

Amongst the *proximate* causes we must especially notice the two following:—

1. Abnormal reciprocal action of the blood and nerves, as well with respect to quantity as to quality. Thus great loss of blood causes faintness and vertigo; determination to the organs of sense gives rise to hallucinations; while different crases of the blood modify the temper.

2. Abnormal associations in the organs of sensation and motion. This includes association between normally separated organs, and the suspension of normal association of organs.

We proceed to notice the arrangement of the psychopathies adopted by our author. They are as follow:—

I. *Folly moria* (*insanity in the more restricted sense*). This form is subject to various differences, according to modifications of the personality, to complication, &c. It may be easily distinguished in practice from fixed delusion, and must not be considered as a higher degree of the latter, a mistake which has occurred, having probably been occasioned, as he supposes, by the circumstance that fixed delusion sometimes passes into folly, and through it into idiocy. It often borders on fixed delusion, and often, by the violence of its outbreaks, on mania.

Craziness and eccentricity (*Trahnwitz una Aberwitz*) are varieties of folly.

II. *Fixed delusion, in its most extensive signification*—the *monomania* of Esquirol. It is equally essential, says our author, what idea governs the patient; whether it concern body or mind; whether it be religious, political, or scientific. The disease consists in this,—that some one idea is able to govern him. The following are the most frequent varieties, arranged according to the objects of the fixed delusion:—

1. Fixed delusion referring to the patient's own body and personality, as when men suppose themselves to be pregnant.

"The cases in which patients imagine that they have toads, frogs, serpents, nay, even men on horseback in their insides; where they are fearful of passing urine, lest they should overflow the town; where they fancy that they have noses of wax or glass, feet of straw, and the like. A woman would not bend her middle finger, because she fancied the world was supported on it. Many patients imagine that they are transformed into an inanimate body, or wholly or in part into another man, or that they unite in themselves two individuals, one in health and the other diseased, and so forth."

2. Of the more psychical fixed ideas, those of ambition are the most frequent; with which is generally connected the idea of being persecuted by all the world. All lunatic asylums abound in kings, popes, prophets, and even sons of God.

3. Next in frequency to this fixed delusion, and often blended with it, is the religious; when the patient considers himself to be a saint exposed to martyrdom. The manifestations of this variety are extremely various. There may be profound melancholy, combined with deep contrition for past sins, (usually of no real existence;) or there may be joyous ecstasy, accompanied by the illusory feeling of special sanctification and Divine grace, heavenly visions, &c. Such cases are too common to require a more detailed notice.

4. Next in order we must place the insanity of love, *erotomania*. The loved object in these cases being often imaginary. In nervous females the delusion often assumes a religious tint, and in this way may be explained the examples of a mystically exalted love, which seems especially frequent in women of the Roman-catholic religion.

5. The fixed delusion that life, either by compulsion or necessity, must be quitted—suicidal monomania. We cannot help extracting the following observations on the suicidal habits of our countrymen:—

"Sometimes it (suicide) is merely caused by the impulse of imitations, as in the Spleen Club, established in England, when two members annually had the right to put an end to their existence; or, as in the case of that beam which ran across one of the bye-streets in London, and offered such convenience for hanging, that some individuals daily suspended themselves from it, till it was at length removed by the police."—P. 282.

It is to be regretted that the learned English editor has not given us any information regarding this very inviting beam; we suspect that the story is better known in Germany than at home.

6. With regard to objects of knowledge, which sometimes serve as matter for fixed delusion, Feuchtersleben concurs in the view taken by Ideler, that this form may always be reduced to the others, especially to that which originates in ambition. So long as the desire for knowledge remains pure, it never can become a delusion. "When any one has lost his reason on the subject of the quadrature of the circle, perpetual motion, &c., it is not the search after truth that has decayed his understanding, for, as Ideler justly observes, he had none to lose."

III. *Madness* (in the most extensive sense, *mania*) originates proximately in an abnormal motor exaltation. *Mania sine delirio* (*manie instinctive sans delire*), which was first described by Pinel, is a variety of this form. "Its essential character is mainly that of mania, a perverse behaviour, which, as Esquirol justly observes, is never wholly automatic, but is always excited by a conception, whether psychical or referring to physical life." Propensities to steal, and to commit infanticide and incendiarism afford illustrations of this form of disease. Our author well observes that this should be termed *delirium in agendo* (*actionum*), rather than *mania sine delino.*

IV. *Idiocy, fatuitas,* proceeds proximately from *anæsthesia,* weakness of attention, and want of images; represents an approximation of the human character to that of animals, and is characterized by an incapacity of judging, or even, in its higher degree, of contemplating. It is divisible into three stages, (1,) stupidity; (2,) idiocy, in the strict acceptation of the word, presenting a total incapacity for mental activity; and, (3,) cretinism.

Under one or other of these forms, or under two combined, our author ranges all psycopathies.

We have yet a few words to say on prognosis, before finishing this portion of the work.

The following are the leading points on which we must base our opinion in individual cases, but we should ever be cautious in our judgment, and "often leave it to a higher power to decide whether a fettered mind be laden with the curse never again to awaken in this mortal life."

We next take into consideration:

1. *Personality in all its relations,* as, for instance, temperament, sex, age, employment, and mode of life, previous education, power of self-control in health, and, above all, hereditary tendency.

2. *The causes of psychoses.*

3. *Their course and duration.* First attacks afford a better prognosis than where they are repeated; psychoses which are rapidly developed a better prognosis than those which increase slowly; and, as a general rule, the hope of cure is in an inverse ratio to the direction of the malady.

Feuchtersleben states as the result of his own experience, that the intermittent form gives a more favourable prognosis than the permanently remittent; many authors assert the contrary.

4. *The complication with other diseases.* The more psychoses are complicated, the more difficult are they to cure. Epilepsy (especially when it precedes the psychoses) and paralysis, are the most unfavourable complication. Deep-seated cachexies are also unfavourable.

5. Lastly, we must be influenced by *external circumstances in so far as they facilitate or impede the execution of a complete plan of cure.* Lunatics, says our author, can scarcely ever hope for cure at home, but only in well organized and well conducted institutions. This is the reason why lunatics of the lower classes are, in general, more curable than those of the higher. They are sent to an asylum at a comparatively early stage of the disease; and are, further, less susceptible of many impressions which impede cure.

With reference to the prognosis of the several forms of the psychoses, it may be observe that mania, particularly if transitory, affords the best prognosis; and if well treated, it leaves behind it the fewest unfavourable consequences. Maniac criminals, according to Ideler, are generally incurable:—"They cannot bear the torments of their consciences, and relapse into the stupefaction of insanity to flee from the consciousness of guilt."

The form of folly is difficult of cure, and its prognosis is especially unfavourable when hallucinations of several senses occur simultaneously. If it occur only once, and then as a sequence to a fit of furious madness, it may be considered favourable, as being an indication of salutary subsidence.

The prognosis in cases of fixed delusion, especially in melancholy, is always unfavourable. Cases of religious delusion are very difficult of cure; so, likewise, are cases of ambitious delusion: a combination of these, which frequently occurs, must be deemed extremely unpropitious.

Idiocy affords the worst prognosis of all. If it be primary, says our author, it indicates mental and bodily imbecility, which cannot be easily removed. If secondary, as the result of another form, it presents a last event, which admits of no further change. Pinel mentions cases of acquired idiocy, in which the mental faculties returned, with alternating fits of mania.

Mania, then, is the most easy of cure; fixed delusion more difficult; folly still more so; and idiocy the most difficult of all.

Having proceeded thus far with our analysis, we must defer, until our next number, the consideration of the therapeutic and judicial portions of the work.

